# Influence of laboratory animal hosts on the life cycle of *Hyalomma marginatum* and implications for an *in vivo* transmission model for Crimean-Congo hemorrhagic fever virus

**DOI:** 10.3389/fcimb.2013.00039

**Published:** 2013-08-20

**Authors:** Aysen Gargili, Saravanan Thangamani, Dennis Bente

**Affiliations:** ^1^Galveston National Laboratory, University of Texas Medical BranchGalveston, TX, USA; ^2^Department of Microbiology and Immunology, University of Texas Medical BranchGalveston, TX, USA; ^3^Faculty of Health Sciences, Marmara UniversityIstanbul, Turkey; ^4^Department of Pathology, University of Texas Medical BranchGalveston, TX, USA

**Keywords:** Crimean-Congo hemorrhagic fever, *Hyalomma marginatum*, BSL4, transmission, Crimean-Congo hemorrhagic fever virus, bunyavirus, tick, tick-borne virus

## Abstract

Crimean-Congo hemorrhagic fever virus (CCHFV) is one of the most geographically widespread arboviruses and causes a severe hemorrhagic syndrome in humans. The virus circulates in nature in a vertebrate-tick cycle and ticks of the genus *Hyalomma* are the main vectors and reservoirs. Although the tick vector plays a central role in the maintenance and transmission of CCHFV in nature, comparatively little is known of CCHFV-tick interactions. This is mostly due to the fact that establishing tick colonies is laborious, and working with CCHFV requires a biosafety level 4 laboratory (BSL4) in many countries. Nonetheless, an *in vivo* transmission model is essential to understand the epidemiology of the transmission cycle of CCHFV. In addition, important parameters such as vectorial capacity of tick species, levels of infection in the host necessary to infect the tick, and aspects of virus transmission by tick bite including the influence of tick saliva, cannot be investigated any other way. Here, we evaluate the influence of different laboratory animal species as hosts supporting the life cycle of *Hyalomma marginatum*, a two-host tick. Rabbits were considered the host of choice for the maintenance of the uninfected colonies due to high larval attachment rates, shorter larval-nymphal feeding times, higher nymphal molting rates, high egg hatching rates, and higher conversion efficiency index (CEI). Furthermore, we describe the successful establishment of an *in vivo* transmission model for CCHFV in a BSL4 biocontainment setting using interferon knockout mice. This will give us a new tool to study the transmission and interaction of CCHFV with its tick vector.

## Introduction

Crimean-Congo hemorrhagic fever (CCHF) is an acute, tick-borne zoonosis caused by Crimean-Congo hemorrhagic fever virus (CCHFV), which belongs to the genus Nairovirus in the family *Bunyaviridae*. Since 2000, the incidence and geographic range of confirmed CCHF cases have markedly increased, with the disease being reported for the first time in Turkey, Iran, India, Greece, the Republic of Georgia, and some Balkan countries, and the detection of viral RNA in *Hyalomma* ticks recovered from deer in Spain. The virus circulates in nature in a vertebrate-tick cycle with ticks of the genus *Hyalomma* being the main vectors and reservoirs. Although the tick vector plays a crucial role in the virus life cycle, most of the work has focused on case studies, epidemiological investigations, and field studies of ticks collected from animal hosts. Despite the need to study the interaction between ticks and the virus in a controlled laboratory setting in order to understand how the virus is transmitted and maintained, little has been done (Shepherd et al., 1989; Gonzalez et al., 1991, 1992; Wilson et al., 1991; Dickson and Turell, 1992; Dohm et al., 1996). This is mostly because establishing and maintaining tick colonies is time consuming, and working with ticks in a biosafety level 4 (BSL4) biocontainment setting is challenging. Ideally, a transmission and infection model utilizes laboratory hosts that both support tick development and virus transmission.

Ticks belonging to the genus *Hyalomma* (*H*.) are considered the main vectors of CCHFV. After the first description of CCHF in 1944 in the agricultural steppe areas of Crimea, *Hyalomma marginatum* was immediately suspected when local inhabitants described unusually high numbers of this species as compared with the previous years (Grashchenkov, 1945). Subsequent outbreaks in Bulgaria, China, Yugoslavia, Pakistan, United Arab Emirates, Iraq, and other areas of Russia in the following decades reaffirmed that species of the genus *Hyalomma* were predominant [thoroughly reviewed by Hoogstraal (1979)]. Details of vector competence were tested for the ability to transmit the virus to the next stage or to eggs from infected females and *H. anatolicum, H. marginatum, H. rufipes, H. turanicum*, and *H. truncatum* are reported the ability to acquire the virus in nature and to pass the virus to the eggs or to the next stage in its life cycle (Hoogstraal, 1979).

**Figure 1:**
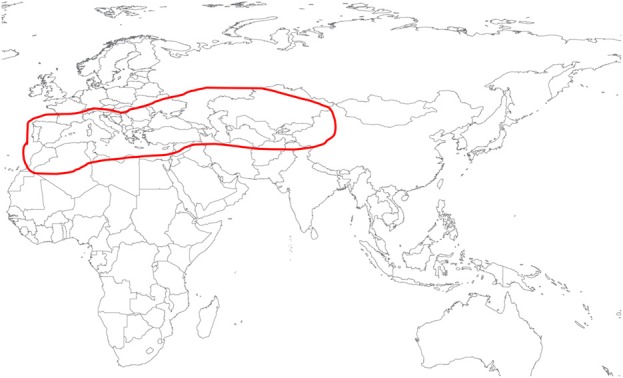
**Geographic distribution of *Hyalomma marginatum* based on (Hoogstraal, 1979), www.kolonin.org and http://www.efsa.europa.eu/en/efsajournal/pub/1723.htm (both accessed April 2013)**.

*H. marginatum* is the most important vector for CCHFV in southern Europe as well as parts of the Middle East and Central Asia (Figure 1). Studies on the life cycle and biology have been published for only 11 of the approximately 27 known *Hyalomma* species (Guglielmone et al., 2010). These studies involve various animal host species in the laboratory maintenance and biology of: *H. aegyptium* (Sweatman, 1968; Siroky et al., 2011). *H. anatolicum* (Snow, 1969; Ghosh and Azhahianambi, 2007; Ahmed et al., 2011), *H. dromedarii* (Bassal and Hefnawy, 1972), *H. excavatum* (Rechav, 1968, 1970; Hadani and Rechav, 1970), *H. impeltatum* (Logan et al., 1989b), *H. isaaci* (Rau, 1963; Das and Subramanian, 1972), *H. lusitanicum* (Hueli et al., 1984a; Ouhelli and Pandey, 1984), *H. rufipes* (Knight et al., 1978; Magano et al., 2000; Chen et al., 2012), and *H. truncatum* (Rechav and Fielden, 1997; Magano et al., 2000).

In nature *H.marginatum* is considered to have a two-host life cycle. This means that larval feeding, molting to nymphal stage, and nymphal feeding will occur on the same host (typically smaller mammals and ground-dwelling birds). Following this, the engorged nymphs drop off from the host, molt to their adult stage in the environment and attach to a second host (typically larger mammals such as ungulates) (Apanaskevich, 2004). Nevertheless, the biology of *H. marginatum* has been poorly investigated (Hueli et al., 1984b; Ouhelli, 1994; Yukari et al., 2011) and limited studies have investigated the life cycle involving domestic animals such as rabbits, cattle, and chickens. There is an urgent need to study the interaction of CCHFV with its natural tick vector in a controlled laboratory setting. Hitherto, studies with ticks at BSL4 have not been reported. Safe experimentation with ticks at this level requires appropriate facilities, extensively trained investigators, and thoroughly tested protocols.

Here, we report our evaluation of three common laboratory species as hosts for *H. marginatum* and their influence on the biology of the tick. Furthermore, we describe for the first time the use of ticks in a BSL4 setting and describe the development of protocols to safely carry out this work.

## Materials and methods

### Ticks

Unfed female and male *H. marginatum* ticks were collected by Dr. Zati Vatansever in Yozgat region of Turkey and species identification was confirmed based on the latest taxonomical key (Estrada-Peña et al., 2004). These ticks were fed on a rabbit, the female tick engorged, dropped off and laid eggs. Female carcass, as well as a sample of the larval offspring, tested negative for CCHFV by RT-PCR (Midilli et al., 2007). Subsequently, larval and nymphal offspring from this female were fed on rabbits and a portion of the emergent adult ticks were sent to the Insectary Services Division of the Galveston National Laboratory, UTMB, Galveston, Texas USA to initiate a colony. Adult ticks were fed on rabbits and first generation larvae hatched from a single female were used for the subsequent experimental studies. Ticks were kept in sterile clear plastic sample vials. Six 2 mm holes cut into the lightweight plastic lid, coupled with a piece of fine mesh approximately 4 cm^2^ in size, served as a secure tick barrier while allowing sufficient air exchange. A folded narrow strip of autoclaved filter paper was maintained inside the vial to absorb excess moisture and allow the ticks to crawl upwards after molting. Ticks were kept under constant conditions (27°C ± 1°C, 80% ± 5% relative humidity, and 12/12 h light/darkness cycle) in the plastic vials within plastic desiccators containing saturated salt solution in the desiccator basin to provide correct humidity levels in environmental growth chambers with temperature and light control. Engorgement of the larvae, nymphs, and adult stages in the following experiments were monitored and ticks that dropped off were collected and weighed daily. Pre-oviposition and oviposition times were recorded for each engorged female. Oviposited egg masses were weighed daily. After the oviposition ended, total egg mass weight for each female and carcasses weight were recorded. Conversion efficiency indexes (CEI = g eggs/g female), which indicates the successful conversion of engorged body weight in to egg mass, were calculated according to Drummond and Whetstone (1970). Unfed nymphs used in the *in vivo* transmission models were generated by pulling off the engorged larvae from the host and then maintaining them under the above described conditions until they molted. All values were compared statistically by ANOVA (GraphPad Prism). Unfed nymphs used in the *in vivo* feeding models were generated by pulling off the engorged larvae from the first host and then maintaining them under the above described conditions until they molted.

### Evaluation of tick killing with disinfectants

Disinfectant solutions commonly used in biocontainment laboratories such as bleach (sodium hypochloride, 5%; Clorox, Oakland, CA), neutral-buffered formalin (10%; Fisher Scientific, MI), ethanol (70%; Sigma-Aldrich, St. Louis, MO), CaviCide® (Metrex Research Coorperation, Romulus, MI), and Microchem Plus® (National Chemical Laboratories, Philadelphia, PA) were tested for their ability to kill adult ticks. Ticks, 8 in each test group, were submerged in 50 ml of each solution and monitored at 15 min intervals. To confirm death, non-motile individuals were taken out of the solution, rinsed with distilled water, wiped down, and placed in the incubator at 27°C, 80% RH for 72 h. Individuals showing any movement after 72 h were recorded as still viable.

### Animals

Female BALB/c mice, 8–12 weeks of age and >20 grams, female Hartley guinea pigs, >65 g, and male New Zealand white rabbits, >1.5 kg, were purchased from a commercial breeder (Charles River, Wilmington, MA) and used to feed instars of *H. marginatum*. None of the animals had been exposed to ticks previously. Mice were housed in isolator cages (Tecniplast, Buguggiate, Italy) with commercial diet and water *ad libitum* and exposed to ticks by either whole body infestation or using feeding capsules (see below). Rabbits and guinea pigs were housed in Allentown isolator cages with commercial diet and water provided *ad libitum*. Rabbits were infested with ticks using ear-bags while guinea pigs were infested with ticks in a feeding capsule (see below). All animal work was reviewed and approved by UTMB's Institutional Animal Care and Use Committee and Institutional Biosafety Committee.

### Tick infestations

- Whole Body Infestation of Mice: Three mice were placed individually into a restrainer made of metal mesh cloth that restricts the mouse from grooming. Three hundred larvae were allowed to attach to the restrained mouse during a 2 h period. After infestation, each mouse was placed into a micro-isolator cage, one mouse per isolator. The micro-isolator cage contained an inner floor consisting of a stainless steel grid above water, allowing the mouse to stay dry and move and feed normally while engorged ticks drop into the water where they were recovered. Water in the bottom of the cage was inspected for ticks and changed daily.
- Feeding Capsule Infestation of Mice: Mice were sedated by isoflurane inhalation (2–4%) and their eyes treated with ointment to prevent drying, and placed on a Delta warming pad (BrainTree sciences). A feeding capsule was fashioned from the top half of a 5 ml cryotube (Sarstedt, Germany). Kamar adhesive (Kamar Products, Zionville, IN) cement (an adhesive commonly used in the livestock industry for maximum adhesion of heat detector pads and tags) applied to the capsule rim. The capsule was attached to a shaved area on the back of the mouse caudal of the shoulder blades. Ticks were placed into the capsule on the following day after the integrity the capsule's attachment was assured by inspection. Adults and nymphs were directly hand-loaded into the capsule using forceps. Larvae were first counted onto to a small piece of gauze or a gelatin capsule, which was then transferred into the capsule. Infestations were done on three mice with 25 larvae in a single capsule, or on five mice with one male-female adult pair in a single capsule. The capsules were inspected daily for engorgement of the feeding instars, and engorged or detached ticks were removed.
- Feeding Capsule Infestation of Guinea Pigs: Guinea pigs were infested with ticks enclosed within a tightly woven cotton cloth fabric feeding capsule. The seam of the bags where the fabric edges join together was sewed tightly using a sewing machine (7 stitches/cm). Animals were sedated by isoflurane gas anesthesia (2–4%). The dorsum was shaved and then a fabric capsule was glued to the skin caudal of the shoulder blades using Kamar glue. Ticks were introduced to the capsule the following day after inspection of the capsule's integrity. The open end of the fabric capsule was folded twice laterally, and then longitudinally (2–3 cm) with the open end secured and was wrapped with multiple layers of adhesive bandages. Three guinea pigs were infested with 200 larvae and another three guinea pigs with three pairs of adults on each animal.
- Ear bag Infestation of Rabbits: Similar to feeding capsules utilized for guinea pigs, ear bags for the rabbit were fabricated using tightly woven cotton cloth. Bags were rectangular in shape with openings at both ends. One open end was attached to the shaved base of the ear with a double layered adhesive bandage and Kamar adhesive. The portion of the bag that extended beyond the length of the ear was folded three times laterally and then longitudinally (2–3 cm), and was wrapped with two layers of adhesive bandages. An Elizabethan collar (Lomir, Biomedical Inc., Malone, NY) was placed on the rabbit and the tips of both ear bags were taped together behind the collar to keep them out of reach and to prevent scratching. Rabbits were sedated with isoflurane gas anesthesia (2–4%) during the application of the ear bags. Eyes were treated with ointment to prevent drying and the rabbit was placed onto a warming pad. Ticks were placed into the ear bags the following day after inspection of the ear bag's integrity. Rabbits were infested with three male-female pairs of adults, or with 300 larvae per ear bag.

### Tick work at BSL4

The development of standard operating procedures (SOPs) for tick work at BSL4 was based on existing SOPs for arthropod containment level (ACL) 2/3 at UTMB as well as on U.S. guidelines (2003; Scott, 2005; Tabachnick, 2006; Prevention, 2009). All work involving infectious material was done in strict compliance with UTMB Environmental Health and Safety guidelines, and Institutional Biosafety Committee and Institutional Animal Care and Use Committee approved guidelines and protocols. For tick work, a simple, minimalist approach was adopted. A room within the BSL4 of the Galveston National Laboratory was designated for tick research and contained only items required for these studies. The room has no sink or floor drain and is equipped with sticky mats on work surfaces, within the glove box, and in front of the door. As described above for colony rearing methods, ticks were contained in capped rearing vials which were housed in sealed desiccators containing appropriate saturated salt solutions to maintain relative humidity, and the desiccators are in turn kept within climate-controlled environmental growth chambers. The researcher must know exactly how many ticks are taken into the facility and account for the numbers at various stages of the experiment through to termination. Accurate counts were maintained throughout the experiments such that records and contents of the rearing vials verified the final state of each tick. Live ticks and ticks feeding on animals were handled within a non-ventilated glove box lined with sticky tape. Since working with arthropods often requires the use of small instruments and considerable dexterity, investigators practiced tick work extensively in the National Biocontainment Training Center mock BSL4 training laboratory while wearing positive pressure suits.

All steps involving the handling of ticks feeding on mice were conducted in a non-ventilated Plexiglas glove box within the BSL4 tick room and animals were sedated using isoflurane inhalation anesthesia. Mice with capsules glued onto the skin were housed individually in green line IVC micro-isolator rat cages (Tecniplast, Buguggiate, Italy), with nestles (Ancare, Bellmore, NY) as enrichment and bright-white Diamond Dry cellulose bedding (Harlan Laboratories, Indianapolis, IN) to easily visualize potentially escaped ticks. Other enrichment items were avoided so that the capsule would not be dislodged by routine contact. As additional layers of containment, a ring of double-sided sticky tape was positioned along the inner perimeter of the cages one inch below the cage top and a mesh cap was placed over the cage air supply ventilation ports to prevent any tick escape from the cage. Ticks in feeding capsules were inspected daily in the glove box and collected from the capsule after detachment and stored in photoperiod controlled environmental chambers for molting to the next stage.

### *In vivo* transmission model

Uninfected *H. marginatum* nymphs or adults were obtained from our established colony and up to five nymphs or up to two adults (male and female) were put into a feeding capsule that had been applied to 4–8 weeks-old female STAT-1 knockout mice (129S6/SvEv-*Stat1^tm1Rds^*; Taconic, Germantown, NY). This mouse strain carries a homozygous disruption of the Signal Transducers and Activators of Transcription 1 gene that eliminates the intracellular mechanism by which cells respond to interferons, resulting in a mouse model with extreme susceptibility to CCHFV (Bente et al., 2010). Animals were moved from Animal Biosafety Level (ABSL) 2 to the ABSL4 3 days after ticks attached and were subsequently challenged intraperitoneally with 100 plaque forming units of CCHFV IbAr 10200 as previously described (Bente et al., 2010). Mice were humanely euthanized on day 4 post challenge when becoming moribund, which allowed sufficient time for all nymphs to complete feeding. Engorged nymphs were allowed to molt to the next stage in plastic vials within plastic desiccators in climate-controlled environmental growth chambers (27°C ± 1°C, 80% ± 5% relative humidity, and 12/12 h light/darkness cycle) within the BSL4 tick room. Before dissection, ticks were euthanized by placing them in a freezer for 30 min. Salivary glands were removed from newly emerged adults under a stereomicroscope. Total RNA was extracted from salivary glands and the remaining tick body by phenol/chloroform extraction with Trizol (Invitrogen, Carlsbad, CA) for RT-PCR testing of CCHFV. Semi-engorged adults were removed 3 days after virus challenge, euthanized, and dissected under the stereomicroscope. Salivary glands, midgut, and ovaries were removed and total RNA isolated from tissues using phenol/chloroform extraction (see above). All samples were tested for CCHFV and amounts calculated as genome equivalence by qRT-PCR using a recombinant RNA standard as previously described (Wolfel et al., 2007) using Qiagen's Quantitect Fast probe kit and were run on a CFX 96 real-time detection system (BioRad, Hercules, CA).

## Results

### Host comparison

**Figure 2:**
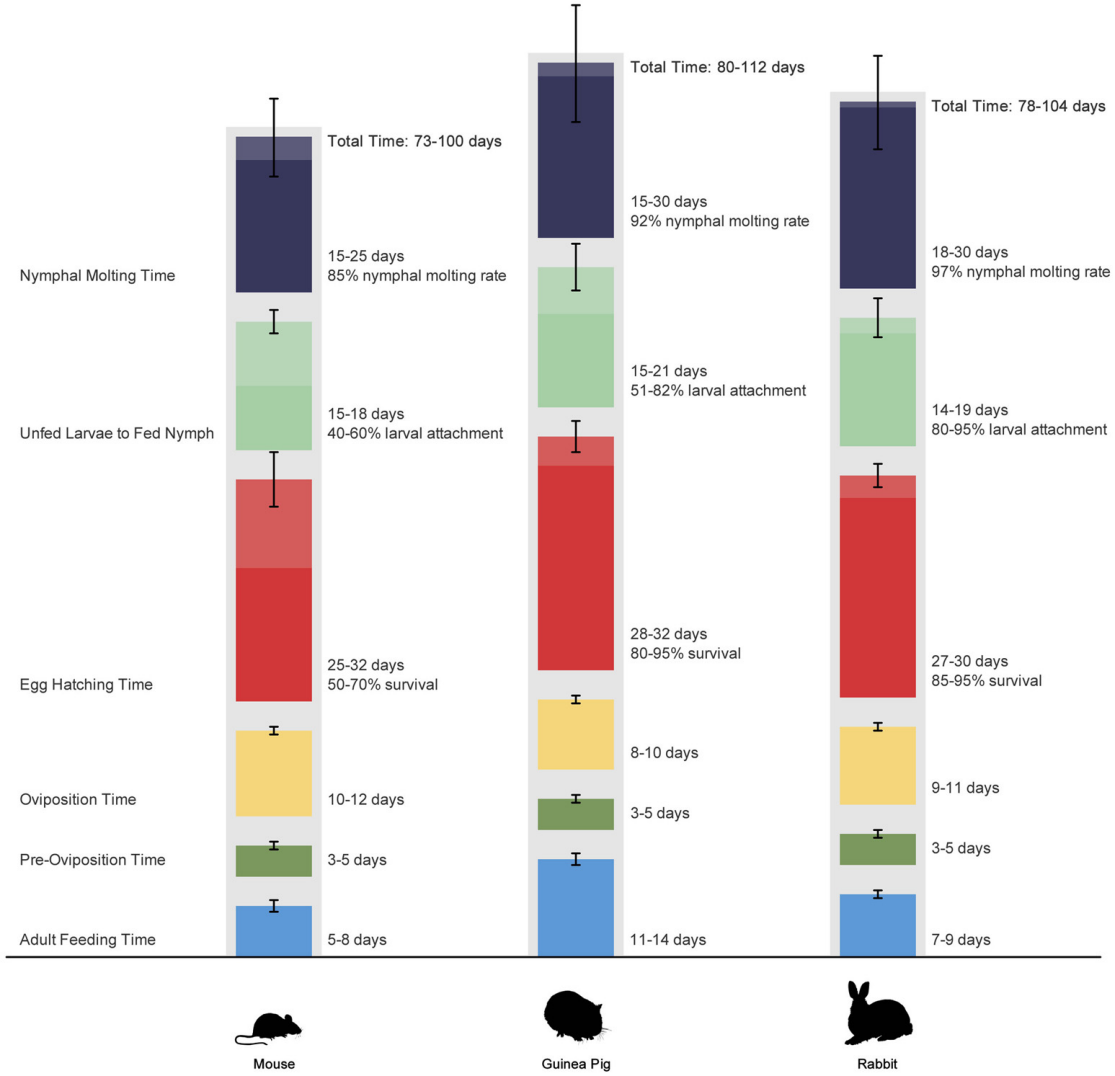
**Life cycle of *Hyalomma marginatum* under laboratory conditions when feeding on mice, guinea pigs, and rabbits**. Colored segments represent time periods required for different life stages. Bars represent range of values. Values were not compared statistically.

In the first part of this study, we evaluated three common laboratory animal species (mice, guinea pigs, and rabbits) as hosts for *H. marginatum* and their influence on the life cycle of the tick. All infestations were done with life stages coming from the same batch and were performed simultaneously on the three hosts to ensure that all ticks were the same age. As seen in nature, *H. marginatum* exhibited a two-host feeding behavior on all three host species (mice, guinea pigs, rabbits) tested with the larval and nymphal stages feeding on the same host. Animals were checked daily after the larval infestation; none dropped off and no crawling engorged larva were observed. The duration of feeding and molting for ticks fed on each host is summarized in Figure 2. The longest time span for the total life cycle was observed for ticks fed on guinea pigs with 112 days and the shortest life cycle was seen in ticks from mice with 73 days. A major difference was seen in adult feeding time among the species tested extending up to 14 days for ticks fed on guinea pigs although the duration of feeding of immature stages appeared similar.

**Figure 3:**
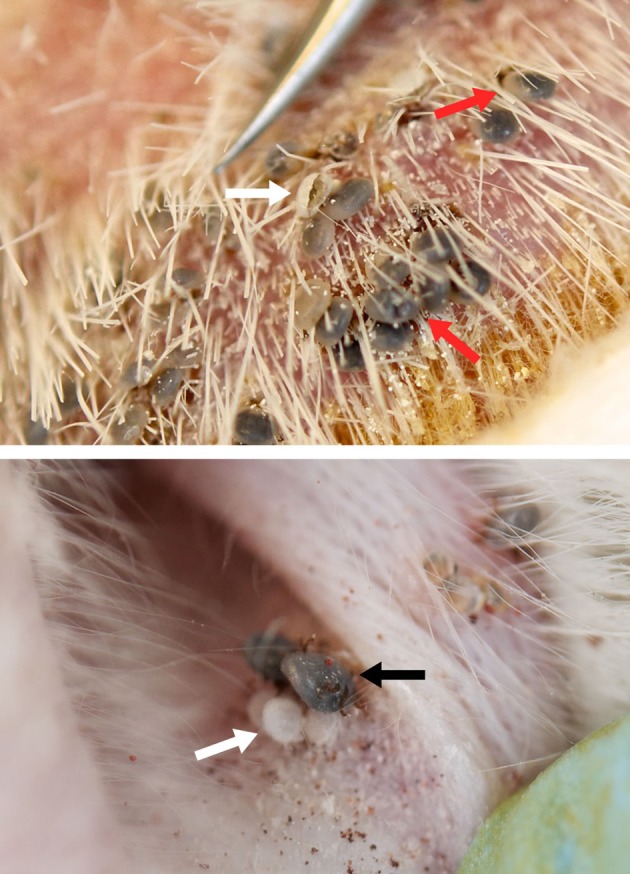
**Molting larvae and newly emerged nymphs feeding on guinea pig (top) and rabbit (bottom)**. Red arrows indicate molting larvae. White arrows show ruptured cuticles left from molted and emerged larvae still attached to the skin. Newly emerged nymphs feeding in close proximity to where they fed as larvae (black arrow).

**Figure 4:**
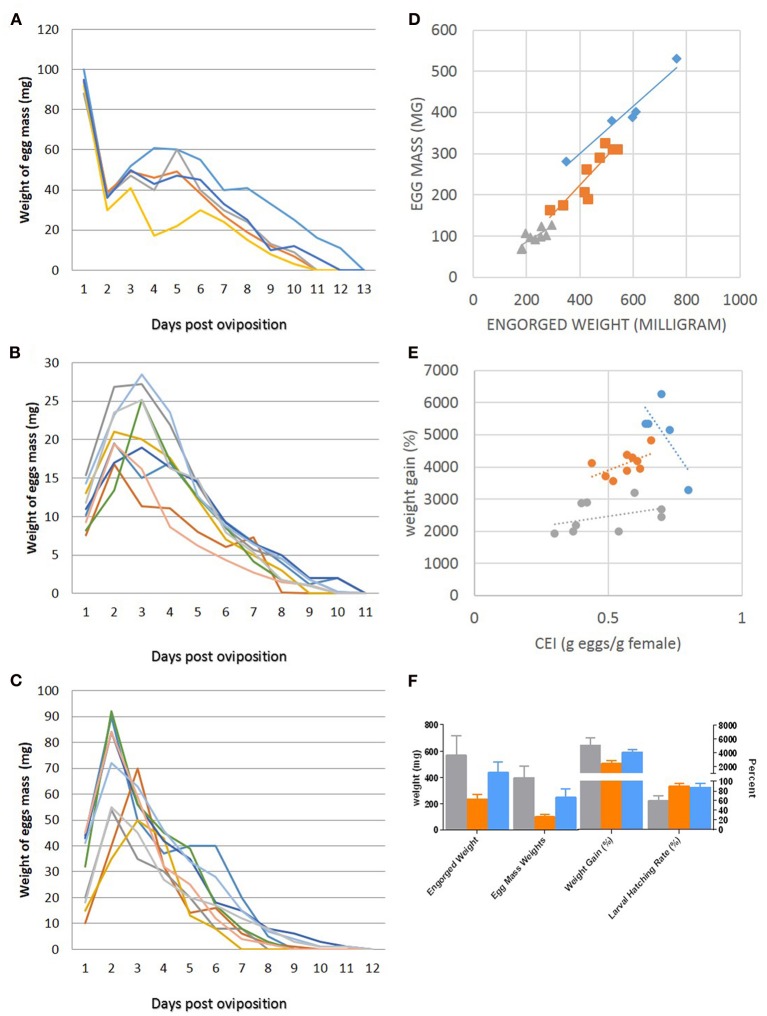
**Summary of oviposition data (A–F)**. Newly deposited egg mass weights measured daily of the females fed on mice **(A)**, guinea pigs **(B)**, and rabbits **(C)**. Each color represents an individual female. Comparisons of egg mass to engorged weight **(D)** and weight gain to conversion efficiency index (CEI) in **(E). (F)** Comparison of the engorged weights, total egg mass weights, and weight gains (%) of the females fed on the three different hosts. For **D–F**, data are color-coded in gray for mice, orange for guinea pigs, and blue for rabbits.

Whole body infestation of mice was the least successful feeding technique among the larval feedings with an attachment rate of 1.55% whereas the larval attachment rates in capsules on mice and guinea pigs and in ear bags on rabbits were 40–60, 51–82, and 80–95%, respectively. As a result of the low attachment success rate during whole body infestation, subsequent studies on mice were conducted using capsule infestations to determine the biological features of *H. marginatum* in mice. Ticks were left on the host molting between larval and nymphal feeding. Larval molting was usually very quick in all tested hosts (2–4 days), and occurred while larvae were still anchored in the host's skin by their mouthparts (Figure 3). The larval exoskeleton ruptured and the newly emerged nymph attached in close proximity to its larval feeding site and then commenced feeding (Figure 3). Mean weights of engorged nymphs that dropped from mice, guinea pigs and rabbits were 20.9 ± 4.6, 13.5 ± 3.9, and 15.25 ± 6.15 mg, respectively and the differences between groups were statistically significant (*p* < 0.05). Molting success rates of engorged nymphs that had fed on either mice, guinea pigs, or rabbits were 85, 92, and 97%, respectively. Molting periods are listed in Figure 2. After molting to the adult stage, male and female ticks were fed on the same host species. Switching host species between different life stages was not attempted in this study. Adult pre-oviposition and oviposition feeding times are shown in Figure 2. Mean engorged weights were 568.20 ± 150.79, 231.88 ± 40.84, and 435.32 ± 83.50 mg for the females that were fed on mice, guinea pigs, and rabbits, respectively and the differences were statistically significant between groups (*p* < 0.05). Oviposited egg masses (mg) of female ticks, representing the total number of eggs laid by an individual female in a 24 h period, feeding on mice, rabbits and guinea pigs were measured daily as depicted in Figures 4 A–C. For females fed on mice, the largest daily egg mass weights were deposited early in the oviposition phase with declining weights over a 10–12 day period; whereas for females fed on guinea pigs and rabbits, oviposition started with lower egg mass weights. Subsequently, the egg mass increased to highest weights at the second and third days and were maintained for 9–11 days in rabbits, and 8–10 days in guinea pigs. Mean total egg mass weights were 396.6 ± 88.84 mg in mice, 98.44 ± 20.38 mg in guinea pig and 248.88 ± 64.93 mg in rabbit. Egg masses were estimated to contain between 2303 and 9288 eggs. The CEI vs. weight can be found in Figures 4D,E. Comparison of the engorged weights, total egg mass weights and weight gains (%) of the females fed on the three different hosts is provided in the Figure 4F. Maximum weight gains were observed in the female *H. marginatum* ticks feeding on mice. Adult *H. marginatum* ticks remove a significant amount of blood during feeding, which could lead potentially lead to anemia in mice, and needs to be taken into consideration when using mice as host model for adult *H. marginatum* ticks. Larvae hatching times are given in Figure 2. Larval hatching rates were between 50–70, 85–95, and 70–90% for the egg batches oviposited by females fed on mice, rabbits, and guinea pigs, respectively.

### Tick control

As part of the establishment of the tick work at BSL4, adult *H. marginatum* ticks were submerged in commonly used disinfectants in order to evaluate the ability to kill ticks (Table 1). Ethanol was fast acting; it killed 100% of the ticks within 1.5 h, followed by formalin and bleach (2 h). Interestingly, Microchem, commonly used as a disinfectant in chemical showers of the BSL4 laboratories, took up to 120 h post exposure to kill 100% of the ticks.

**Table 1:** **Killing times of *Hyalomma marginatum* using different disinfectants**.

**Time**	**Cavicide**	**Microchem 5%**	**Formalin 10%**	**Bleach 5%**	**Ethanol 70%**
15 min	0	0	0	0	0
30 min	0	0	0	0	25
45 min	0	0	0	0	50
60 min	0	0	0	25	75
1.5 h	0	0	75	75	100
2 h	0	0	100	100	
3 h	0	0			
4 h	25	0			
6 h	75	0			
8 h	100	0			
12 h		0			
24 h		0			
36 h		0			
48 h		25			
72 h		50			
96 h		75			
120 h		100			

*Table depicts the percent of ticks that are killed when submerged in disinfectant for a particular amount of time. Experiments were done with eight ticks in each group. Ticks were rinsed with deionized water after exposure, wiped clean, and kept in the incubator for 72 h at 27°C with 80% RH to determine if any ticks become active*.

### Transmission studies

A total of five non-infected adult *H. marginatum* ticks were fed on three STAT-1 KO mice challenged with 100 PFU of CCHFV IbAr 10200 and removed 3 days post virus challenge. Ticks were dissected, RNA extracted from both salivary gland pairs, midgut and ovaries, and were tested were for CCHFV by QRT-PCR. CCHFV RNA was found in pairs of salivary glands (1.01 × 10^7^ to 2.31 × 10^9^ genome equivalents), total midgut (3.74 × 10^4^ to 1.67 × 10^7^ genome equivalents) and in the ovaries (8.54 × 10^2^ to 6.12 × 10^3^ genome equivalents) of all five ticks. Subsequently, a total of 8 CCHFV-negative *H. marginatum* nymphs were fed on 3 STAT-1 KO mice that had been challenged with 100 PFU of CCHFV IbAr 10200. After molting to the adult stage was completed, ticks were dissected and salivary glands were removed. RNA was extracted from salivary glands and remaining tick body. All eight adult ticks were positive for CCHFV by QRT-PCR. The CCHFV genome equivalents values ranges from 8.68 × 10^5^ to 3.05 × 10^8^ in the body and 4.74 × 10^4^ to 6.49 × 10^8^ in the pairs of salivary glands.

## Discussion

*H. marginatum* is considered to be the main vector for CCHFV in southern Europe, parts of the Middle East, Africa, and Central Asia (Figure 1). Only three studies have characterized the life cycle of this tick species in a laboratory setting (Hueli et al., 1984b; Ouhelli, 1994; Yukari et al., 2011) and no studies describe the life cycle of this species when fed on laboratory animals. Studies on the transmission of CCHFV with this tick species are also very limited and involve only the natural hosts (Zgurskaya et al., 1971; Levi and Vasilenko, 1972; Blagoveshchenskaya et al., 1975; Kondratenko, 1976; Zarubinsky et al., 1976). Here we characterized three different laboratory animal species as experimental hosts for *H. marginatum*. The genus *Hyalomma* includes one, two or three host tick species. Some species such as *H. anatolicum, H. scupense*, and *H. dromedarii* can complete their life cycle using either one or two hosts. Another member of the genus, *H. excavatum* is known to follow two or three host biology depending on the hosts (Apanaskevich, 2004). *H. marginatum* is known as a two host tick species, feeding on birds and small mammals as immatures, and feeding as adults on large mammals primarily on artiodactyls (Hoogstraal, 1979; Apanaskevich, 2004). Yukari et al. (2011) reported that *H. marginatum* is a strict two host tick within their experimental design using rabbits and cattle for larval and adult feeding, respectively. Conversely, Ouhelli (1994) suggested that *H. marginatum* is able to switch to a three host life cycle as seen with *H. scupense* and *H. excavatum*. However, only data of a two host life cycle were presented. Furthermore, Ouhelli describes *H. marginatum* adults as not completing their engorgement on the rabbit host. We observed however that *H. marginatum* adults completed feeding successfully on rabbits, confirming the report of Yukari et al. studies (2011). More importantly, we also demonstrated that *H. marginatum* shows a strict two-host life cycle with all three host species: larvae engorged and molted to the nymphal stage onsite and attached to the same host on all three host types.

Capsule feeding is desirable in a biocontainment setting to better control the ticks especially before feeding commences. We expected that the feeding capsule would result in a generally lower attachment rates since a feeding capsule does not give the tick the full opportunity to quest for an appropriate feeding spot. Nonetheless, attachment success rates during capsule feeding were generally high and varied between 40 and 95% depending on the host. Interestingly, whole body infestation rates of larvae on mice was far lower (1.55% success rates) compared to capsule feeding (>40%). Although the lower level of attachment rates might have been due to the removal of ticks during grooming, Hoogstraal proposed that rodents are generally not an appropriate host for immature stages of *H. marginatum* (Hoogstraal, 1979). *H. isaaci*, previously grouped as a subspecies of *H. marginatum* (formerly *H. marginatum isaaci*), was also reported to not parasitize rats and mice experimentally (Das and Subramanian, 1972). In another experimental study, *H. rufipes* (formerly *H. m. rufipes*), was also reported to attach poorly to field mice (Magano et al., 2000). Most rodents live in burrows in nests with considerably higher humidity, which does not fit the environmental needs of *H. marginatum* and other former subspecies. Also, these rodents have a social grooming behavior which is likely to remove a large proportion of instars. All of these factors may have played a role in the development of an incompatibility between these tick species and host species.

Das and Subramanian (1972) reported that 91.5% of *H. isaaci* larvae attached to rabbit and dropped as engorged nymphs in 15–18 days, while very few larvae attached and engorged on guinea pigs. In our study, similar attachment results were seen for *H. marginatum* on rabbits; however, attachment rates on guinea pig were far greater (51–82%).

Molting times for nymphs in our study were similar for all three species studied, and correlate with what has been reported in nature (Petrova-Piontkovskaya, 1947) and in laboratory conditions (Yukari et al., 2011).

Mean engorged weights of nymphs were measured as 20.9 ± 4.6, 15.25 ± 6.15 and 13.5 ± 3.9 mg for those fed on mice, rabbits, and guinea pigs, respectively. Significantly, higher mean engorgement weights were recorded on nymphs of *H. truncatum* which fed on the field mice as well and the authors explained this finding with the feeding on non-natural and natural hosts of the ticks (Magano et al., 2000). Molting rates were 85, 92, and 97% for the nymphs that dropped off from mice, guinea pigs, and rabbits, respectively. Similar molting rates were reported with rabbits (Hueli et al., 1984b). For *H. rufipes* nymphs, higher molting success was reported when engorged on guinea pigs (100%) and striped mice (*Rhabdomys pumilio*) (99.9%). Considering that *H. rufipes* has shown three-host feeding pattern on mice, which is not seen in *H. marginatum* biology, this elevated molting success could have arisen due to the adjustment of this species to hosts of a wider range (Magano et al., 2000).

Adult feeding times (for female ticks) were considerably longer for those fed on guinea pigs (11–14 days) as compared to rabbits (7–9 days), or mice (5–8 days). Feeding times on rabbits were reported to be up to 21 (Yukari et al., 2011) and 13 days (Hueli et al., 1984b) previously. On the other hand, higher engorgement weights were seen in females fed on mice (556 ± 207 mg), although feeding times for this stage on this host were considerably shorter (5–8 days) in our study. Differences seen in feeding time and engorgement weights on various hosts could be explained by factors of feeding on natural and non-natural hosts, in a similar way to that reported for nymphal stages (Magano et al., 2000); however, questions such as why different stages engorge with different maximum weights on different hosts, requires further detailed studies involving immunological aspects of the hosts and feeding patterns.

Daily oviposition by the females fed on different hosts showed distinct patterns as seen in Figures 4A–C. When fed on guinea pigs and rabbits, oviposition started with lower egg mass weights, subsequently, the egg mass increased to the highest weights at 2nd–3rd day as described similarly by Ouhelli (1994) who fed the female ticks on rabbits and cattle. On the contrary, when fed on mice, oviposition began with the highest daily egg mass weights with subsequent egg masses weighing less each day. Considering the cattle and rabbits are the natural hosts for *H. marginatum*, females fed on guinea pigs could be regarded as showing similar egg laying pattern to natural biology.

Here, we also describe our work with ticks at BSL4, which to our knowledge not been reported before. The construction of a BSL4 facility and the arrangement of a tick facility within it, low humidity maintained in the facility and required operating procedures all are sufficient to guarantee that no escaped tick life stage could survive for an extended time. Nevertheless, it remains a fundamental responsibility for those working with ticks in the BSL4 laboratory to ensure that all ticks are safely contained. Arthropod security is based on multiple layers of containment. As demonstrated here, ticks are very resistant to being submerged or covered in disinfectant solution of any kind. Although a tick is most likely to be washed off the positive pressure protective suit during the mandatory chemical decontamination shower when exiting the BSL4 facility—and would ultimately be destroyed during the effluent waste sterilization process—this method of killing the ticks would be considered the worst case scenario. The most critical objective is to take every possible precaution to ensure that all ticks remain accounted for in the dedicated room within the BSL4. We demonstrated in our study that all tick stages can be successfully fed within a feeding capsule fastened to the animal's back, which further facilitates controlling the tick escape. Although the use of a capsule denies the tick's innate behavior to quest for a suitable feeding site, the success rates demonstrated in our studies were high.

In order to study the interaction of CCHFV with its tick host, a technique that reliably infects ticks with the virus is desirable. Parental virus inoculation such as oral or anal are difficult to conduct in instar stages of ticks whereas intracolemic inoculation is not useful for vector competence studies (Randolph and Nuttall, 1994). Furthermore, all above mentioned techniques are difficult to conduct in a BSL4 setting. A host feeding model which mimics the natural infection is therefore desirable. Previous studies looked at CCHFV transmission from host animals challenged with the virus to different species of *Hyalomma* ticks (Logan et al., 1989a; Shepherd et al., 1989; Dickson and Turell, 1992; Gordon et al., 1993; Dohm et al., 1996). Generally speaking, the transmission rates are fairly low. For example, Logan et al. noted an 4.4% tick infection rate when feeding on suckling mice challenged with CCHFV (Logan et al., 1989a). Transmission rates were 13% for *H. impeltatum* nymphs feeding on CCHFV-challenged guinea pigs (Dohm et al., 1996). This is most likely due to the fact that the utilized host animals have no or very low level viremia compared to some of their natural hosts. Interferon response knockout mice have recently been described as an animal model that mimics human disease (Bente et al., 2010; Bereczky et al., 2010; Zivcec et al., 2013). It was demonstrated that CCHFV replication results in viremia levels of up to 10^10^ genome equivalents per ml (Bente et al., 2010) or >10^3^ TCID_50_/μ l (Zivcec et al., 2013). We hypothesized that feeding *H. marginatum* nymphs on STAT-1 KO mice will ensure that the tick is exposed to high levels of CCHFV during feeding, and therefore, result in a reliable infection of the tick with the virus. As hypothesized, the high viremia level resulted in 100% infection of *H. marginatum* nymphs during feeding as well as 100% infection after molting, which demonstrates transstadial transmission. Therefore, the *in vivo* feeding model on STAT-1 KO mice is a means to reliably infect *H. marginatum* ticks with CCHFV. Furthermore, the CCHFV-infected adults that are generated in this model can then be used to study the transmission of CCHFV from the tick to the mammalian host. Despite the limitation of only be able to use small tick numbers per experiment, this is a valuable tool to study the transmission of the virus and vector competence for CCHFV of various other tick species.

### Conflict of interest statement

The authors declare that the research was conducted in the absence of any commercial or financial relationships that could be construed as a potential conflict of interest.
